# INSANet: INtra-INter Spectral Attention Network for Effective Feature Fusion of Multispectral Pedestrian Detection

**DOI:** 10.3390/s24041168

**Published:** 2024-02-10

**Authors:** Sangin Lee, Taejoo Kim, Jeongmin Shin, Namil Kim, Yukyung Choi

**Affiliations:** 1 Department of Software, Sejong University, Seoul 05006, Republic of Korea; silee@rcv.sejong.ac.kr; 2 Department of Convergence Engineering for Intelligent Drone, Sejong University, Seoul 05006, Republic of Korea; tjkim@rcv.sejong.ac.kr (T.K.); jmshin@rcv.sejong.ac.kr (J.S.); 3 NAVER LABS, Seongnam 13561, Republic of Korea; namil.kim@naverlabs.com

**Keywords:** autonomous vehicle, computer vision, data augmentation, feature fusion, multispectral, pedestrian detection

## Abstract

Pedestrian detection is a critical task for safety-critical systems, but detecting pedestrians is challenging in low-light and adverse weather conditions. Thermal images can be used to improve robustness by providing complementary information to RGB images. Previous studies have shown that multi-modal feature fusion using convolution operation can be effective, but such methods rely solely on local feature correlations, which can degrade the performance capabilities. To address this issue, we propose an attention-based novel fusion network, referred to as INSANet (INtra-INter Spectral Attention Network), that captures global intra- and inter-information. It consists of intra- and inter-spectral attention blocks that allow the model to learn mutual spectral relationships. Additionally, we identified an imbalance in the multispectral dataset caused by several factors and designed an augmentation strategy that mitigates concentrated distributions and enables the model to learn the diverse locations of pedestrians. Extensive experiments demonstrate the effectiveness of the proposed methods, which achieve state-of-the-art performance on the KAIST dataset and LLVIP dataset. Finally, we conduct a regional performance evaluation to demonstrate the effectiveness of our proposed network in various regions.

## 1. Introduction

Pedestrian detection, which involves predicting bounding boxes to locate pedestrians in an image, has long been studied due to its utility in various real-world applications, such as autonomous vehicles, video surveillance and unmanned aerial vehicles [1,2,3,4]. In particular, robust pedestrian detection in challenging scenarios is essential in autonomous driving application since it is directly related to human safety. However, modern RGB-based pedestrian detection methods failed to operate in challenging environments characterized by low illumination, rain, and fog [5,6,7,8]. To alleviate this problem, several methods [5,9,10] have emerged that leverage a thermal camera as a sensor complementary to the RGB camera already in use. Thermal cameras offer visual cues in challenging environments by capturing long-wavelength radiation emitted by subjects, thereby overcoming the limitations of RGB cameras in complex conditions.

To achieve successful multispectral pedestrian detection, it is important to consider three key factors: enhancing individual spectral features, understanding the relationships between inter-spectral features, and effectively aggregating these features. Building upon these principles, diverse multispectral pedestrian detection approaches have emerged, including single/multi-scale feature fusion [11,12,13,14,15,16] as well as iterative fusion-and-refinement methods [17,18]. These approaches have achieved impressive results with novel fusion techniques. However, most previous methods rely on a convolutional layer to enhance the modality-specific features and capture the correlations between them. Due to the lack of a receptive field in such convolution layers given their small kernel size, they have trouble capturing the long-range spatial dependencies of both intra- and inter-spectral images.

Recently, transformer-based fusion methods [19,20] that enhance the representation of each spectral feature map to improve the multispectral feature fusion have emerged. These methods capture the complementary information between multispectral images by employing an attention mechanism that assigns importance to input sequences by considering their relationships. While existing approaches achieve satisfactory detection results, they still have the disadvantage of neglecting or inadequately addressing the inherent relationship among intra-modality features.

**Figure 1:**
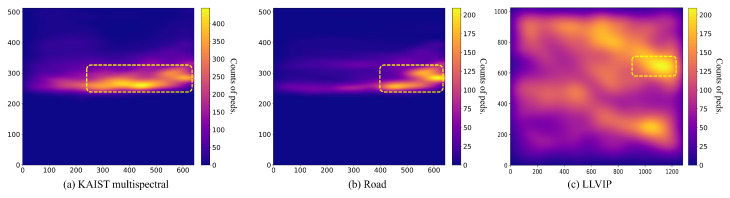
Analyzing the distribution of pedestrians in the KAIST multispectral dataset and LLVIP dataset using Gaussian Kernel Density Estimation (Gaussian KDE). In the (**a**) KAIST dataset, especially in the (**b**) road scene, pedestrians are more concentrated on the right side of the image for several reasons, including the road environment, where sidewalks are clearly divided and a right-hand driving condition prevails. In the (**c**) LLVIP dataset, while displaying a more uniform distribution, there is a persistent bias toward pedestrian over-appearance on the right side of the images. A plasma colormap is used to encode the distribution of the density, with blue indicating low density and yellow indicating high density. High density is marked with a yellow square.

In addition, we observed that the detection performance was restricted due to the imbalanced distribution of locations where pedestrians appear. This imbalanced distribution frequently occurs in both multispectral [5,10] and single-spectral thermal pedestrian detection datasets [21,22]. To analyze this phenomenon, we plot the distribution of the center of annotated pedestrians in the KAIST multispectral dataset and LLVIP dataset in Figure 1. As shown in the yellow square in Figure 1a, the number of pedestrian appearances is concentrated in specific regions biased to the right side. This result stems from the fact that KAIST dataset entries are acquired under right-hand traffic conditions, making it challenging to provide sufficient sight to detect pedestrians on the left side. In particular, pedestrian counts become intensely imbalanced in road scenarios where images were collected along arterial roads where sidewalks and traffic lanes are sharply divided (as shown in Figure 1b). As observed in Figure 1c, the phenomenon of pedestrian concentration persists even though the LLVIP dataset was captured from a video surveillance camera angle. To mitigate the distribution imbalance, it is a common practice to employ standard geometric data augmentations such as cropping and flipping. However, even when applying these data augmentation methods, we found that the over-appearance problem persisted in some regions.

This paper presents a comprehensive study of a method to improve the performance of a multispectral pedestrian detection framework by addressing the issues described above. We propose a novel fusion module, INtra-INter Spectral Attention, which consists of intra- and inter-modality attention blocks that effectively integrate complementary information across different spectral modalities. Specifically, the intra-modality attention block performs the self-attention within each modality feature map to suppress irrelevant information, effectively enhancing modality-specific information. These enhanced feature maps encourage the inter-modality attention block to calculate the mutual relationships between cross-modalities to improve the multispectral feature fusion outcome. We also analyze standard geometric transformations to address the imbalanced distribution of pedestrian locations in the training data. As a result, we find that shifting the image along the x-axis within a specific range mitigates the over-representation of pedestrians in certain regions. Our method achieves state-of-the-art performance on the KAIST multispectral pedestrian detection dataset and LLVIP dataset, demonstrating the effectiveness of our contributions.

## 2. Related Work

### 2.1. Multispectral Pedestrian Detection

Multispectral pedestrian detection research has made significant progress with thermal images able to detect accurately pedestrians in a variety of challenging conditions. Hwang et al. [5] released a large-scale multispectral pedestrian dataset and proposed a hand-crafted Aggregated Channel Feature (ACF) approach that utilized the thermal channel features. This work had a significant impact on subsequent multispectral pedestrian detection research. Liu et al. [23] analyzed the feature fusion performance outcomes at different stages using the NIN (Network-In-Network) fusion strategy. Li et al. [16] demonstrated that multi-task learning using semantic segmentation could improve object detection performance capabilities compared to a detection-only approach. Zhang et al. [17] proposed a cyclic multispectral feature fusion and refinement method that improves the representation of each modality feature. Yang et al. [24] and Li et al. [25] designed an illumination-aware gate that adaptively modulates the fusion weights between RGB and thermal features using illumination information predicted from RGB images. Zhou et al. [18] leveraged common- and differential-mode information simultaneously to address modality imbalance problems considering both illumination and feature factors. Zhang et al. [11] proposed a Region Feature Alignment (RFA) module that adaptively interacts with the feature offset in an effort to address weakly aligned phenomena. Kim et al. [15] proposed a novel multi-label learning method to distinguish between paired and unpaired images for robust pedestrian detection in commercialized sensor configurations such as stereo vision systems. Although previous studies have achieved remarkable performance gains, convolution-based fusion strategies struggle to capture the global context effectively in both intra- and inter-spectral images despite the importance of doing so during the feature fusion process. To address this issue, we design a transformer-based attention scheme in this paper.

### 2.2. Attention-Based Fusion Strategies

Attention mechanisms [26,27,28] have led to a model capable of learning enhanced modality-specific information. Zhang et al. [12] proposed a cross-modality interactive attention mechanism that encodes the interaction between RGB and thermal modalities and adaptively fuses features to improve the pedestrian detection performance. Fu et al. introduced a pixel-level feature fusion attention module that incorporates spatial and channel dimensions. Zhang et al. [13] designed Guided Attentive Feature Fusion (GAFF) to guide the feature fusion of intra-modality and inter-modality features with an auxiliary pedestrian mask. With the success of the attention-based transformer mechanism [29] in natural language processing (NLP) and the subsequent development of a vision transformer (ViT) [30], several methods have attempted to utilize transformer-based attention schemes for multispectral pedestrian detection. Shen et al. [20] proposed a dual cross-attention transformer feature fusion framework for simultaneous global feature interaction and complementary information capturing across modalities. The proposed framework uses a query-guided cross-attention mechanism to interact with cross-modal information. Zhu et al. [31] proposed a Multi-modal Feature Pyramid Transformer (MFPT) using a feature pyramid architecture that simultaneously attends to spatial and scale information within and between modalities. Fang et al. [19] leveraged self-attention to execute intra-modality and inter-modality fusion simultaneously and to capture the latent interactions between RGB and thermal spectral information more effectively. However, transformer-based feature fusion methods have not yet fully realized the potential of attention mechanisms, as they do not effectively learn the complementary information between modalities. In this paper, we propose an effective transformer-based module that enhances and interacts with intra- and inter-specific information.

### 2.3. Data Augmentations in Pedestrian Detection

Data augmentation is a key technique for improving the robustness and generalization of object detection. Pedestrian detection models commonly use augmentation approaches such as geometric transformations, including flips, rotation, and cropping, as well as other techniques such as zoom in, zoom out, cutmix [32], mixup [33], and others. In a previous study, Cygert et al. [34] proposed patch-based augmentation that utilized image distortions and stylized textures to achieve competitive results. Chen et al. [35] proposed shape transformations to generate more realistic-looking pedestrians. Chi et al. [36] and Tang et al. [37] introduced an occlusion-simulated augmentation method that divides pedestrians into parts and fills in with the mean values of ImageNet [38] or images to improve the degree of robustness to occlusions. To address the motion blur problem in an autonomous driving scene, Khan et al. [39] designed hard mixup augmentation, which is an image-aware technique that combines mixup [33] augmentation with hard labels. To address the paucity of data on severe weather conditions, Tumas et al. [40] used a DNN-based augmentation that modified training images with Gaussian noise to mimic adverse weather conditions. Kim et al. [15] proposed semi-unpaired augmentation, which stochastically applies augmentation to one of the multispectral images. This breaking of the pair allows the model to learn from both paired and unpaired conditions, demonstrating good generalization performance. In this paper, we propose a simple yet effective shift augmentation method that disperses peak regions in the image, allowing the model to learn from a variety of regions.

## 3. Materials and Methods

This section presents a comprehensive study on multispectral pedestrian detection. First, we describe the overall architecture of our detection network and our novel INtra-INter Spectral Attention module. We also introduce an effective data augmentation methods, the shift augmentation technique, to address the imbalanced distribution of pedestrian locations. Details about the architecture design and shift augmentation method are provided in Section 3.1 and Section 3.2, respectively.

### 3.1. Model Architecture

#### 3.1.1. Overall Framework

**Figure 2:**
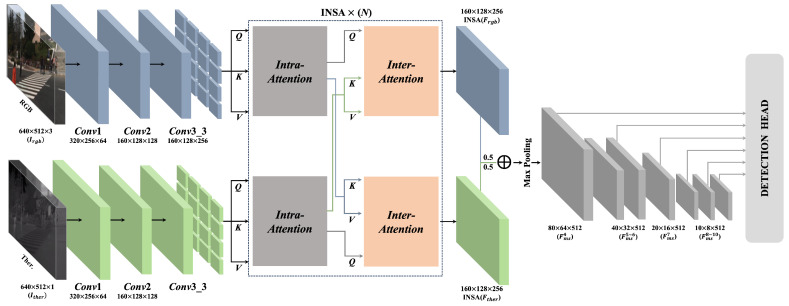
Overall framework of the proposed network: INtra-INter Spatial Attention Network (INSANet). $F_{rgb}$, $F_{ther}$ and $f_{ms}^{i}$ indicate RGB, the thermal feature map and the *i*-th merged feature map, respectively. Q, K, and V correspondingly indicate query, key, and value. After passing through the INSA module, $F_{rgb}$ and $F_{ther}$ are merged by weighted summation (α == 0.5).

The key concern when undertaking robust multispectral pedestrian detection is to integrate complementary information from different spectral images properly. In this aspect, many researchers have adopted a halfway-based architecture that extracts modality-specific features from the intermediate layers of convolutional neural networks and interacts with multispectral feature maps before forwarding them to the detection heads. Similar to how halfway-based fusion methods work, the model proposed here consists of three major parts (Figure 2): (1) modality-specific feature extractors, (2) an INtra-INter Spatial Attention (INSA) module for multispectral feature fusion, and (3) an auxiliary network followed by detection heads. Note that the INSA module and auxiliary network are weight sharing, contrary to modality-specific feature extractors. This weight-sharing design encourages the INSA module and auxiliary network to facilitate the integration of complementary information between multispectral features. On the other hand, two independent feature extractors without weight sharing explicitly consider the modality-specific information.

Specifically, $f_{rgb}$ and $f_{ther}$ represent the RGB and thermal feature extractors, respectively. These extractors take an RGB image and a thermal image as input, respectively, and extract modality-inherent feature maps that are down to one-quarter of the original resolution as follows:

(1) $$\begin{array}{cc} \; & F_{\theta }=f_{\theta }\left(I_{\theta }\right),\;\theta \in \left\{rgb,ther\right\} \end{array}$$

Here, $F_{\theta }$ and $I_{\theta }$ refer to the modality-specific feature map and the image, respectively. After feature extraction, these independent features are fed to our INSA module, which consists of self-attention and cross-attention, followed by the feed-forward network. This module enhances feature representation within each modality while also facilitating exchanges of complementary information between modalities. A detailed explanation of the INSA module is given in Section 3.1.3. After passing through the INSA module, the enhanced spectral feature maps are merged by weighted summation. This fused feature map is then subjected to maxpooling to form a multispectral feature map.

(2) $$\begin{array}{cc} \; & F_{ms}=maxpool\left(\alpha {\hat{F}}_{rgb}⊕\left(1-\alpha \right){\hat{F}}_{ther}\right)\in R^{\frac{H}{8}\times \frac{W}{8}\times C},\;\alpha ==0.5 \end{array}$$

where ${\hat{F}}_{rgb}$ and ${\hat{F}}_{ther}$ are enhanced RGB and thermal feature maps through proposed INSA module, respectively. Finally, the auxiliary network takes the merged feature map ($F_{ms}$) and generates multi-scale feature maps $F_{ms}^{N}$ through a series of convolutional and pooling layers. These multi-scale feature maps are then passed to the detection heads, which consist of two separate convolution layers for classifying and localizing the pedestrians. Our detection network reduces the computational cost and number of trainable parameters by directly passing the first merged features through the modality-sharing auxiliary network and detection heads.

#### 3.1.2. Attention-Based Fusion

##### Preliminary Transformer-Based Fusion

We briefly introduce the attention mechanism of the transformer, which is a powerful technique that calculates the relationships among input sequences. The attention mechanism can be implemented as follows:

(3) $$\begin{array}{cc} Attn(X_{a},X_{b}) & =softmax\left(\frac{X_{a}W_{Q}\cdot W_{K}^{T}X_{b}^{T}}{\sqrt{d}}\right)\cdot X_{b}W_{V}\\ Cross-Attn(X_{a},X_{b}) & =Attn\left(X_{a},X_{b}\right),\;Attn\left(X_{b},X_{a}\right)\\ Self-Attn(X_{a}) & =Attn(X_{a},X_{a}) \end{array}$$

where $W_{Q}$, $W_{K}$ and $W_{V}$ are learnable parameters that project the input token to query, key, and value, respectively. *X* and *d* correspondingly indicate the input token and length of the query dimension. In other words, the attention mechanism calculates the attention weights between the query and key using inner products and then applies softmax to scale the attention weights. Finally, the attention weights are applied to the value matrix. This mechanism helps the model focus on the most relevant parts of the input data.

In multispectral pedestrian detection, the aforementioned attention mechanism can be leveraged to consider the complementary information between multispectral images, as follows:

(4) $$\begin{array}{c} {\hat{X}}_{rgb},\;{\hat{X}}_{ther}=Cross-Attn\left(X_{rgb},X_{ther}\right) \end{array}$$

where ${\hat{X}}_{rgb}$ and ${\hat{X}}_{ther}$ are the enhanced RGB and thermal feature maps, respectively, as determined by calculating the correlation among multispectral features. Shen et al. [20] proposed the following cross-modal feature-enhanced module that modifies the equation above:

(5) $$\begin{array}{cc} \; & {\hat{X}}_{rgb},\;{\hat{X}}_{ther}=upsample\left(Cross-Attn\left(pool\left(X_{rgb}\right),pool\left(X_{ther}\right)\right)\right) \end{array}$$

In Equation (5), pool and upsample indicate the pooling operations for downsampling and upsampling, respectively. Because the attention mechanism has quadratic computation complexity to the input resolution, they applied a pooling operation before calculating the attention weight to reduce the computation cost. However, cross-attention-based feature enhancement may sacrifice a potential performance gain because it neglects to capture the context within each modality.

In another approach, concatenated self-attention (CatSelf-Attn), which undertakes the self-attention of concentrated multispectral features along the spatial axis, was developed.

(6) $$\begin{array}{cc} CatSelf-Attn & =upsample(Self-Attn\left(X_{ms}\right)),\\ X_{ms} & =Cat(pool\left(X_{rgb}\right),\;pool\left(X_{ther}\right)) \end{array}$$

CatSelf-Attn can simultaneously aggregate intra-modality and inter-modality information, but the computational complexity grows quadratic with an increase in the number of input tokens involved in the attention operation. Furthermore, because excessive pooling is applied to feature maps prior to entering the previous feature enhancement modules for computational efficiency, these modules face the challenge of insufficient feature representation during the attention calculation.

#### 3.1.3. INtra-INter Spectral Attention

The main concerns when designing a feature enhancement module are as follows: computing relationships within each modality and across modalities, and balancing the trade-off between computational efficiency and information loss. With regard to these consideration, we propose the INtra-INter Spectral Attention (INSA) module, which enhances the representation of each spectral feature map through a combination of intra- and inter-spectral attention.

**Figure 3:**
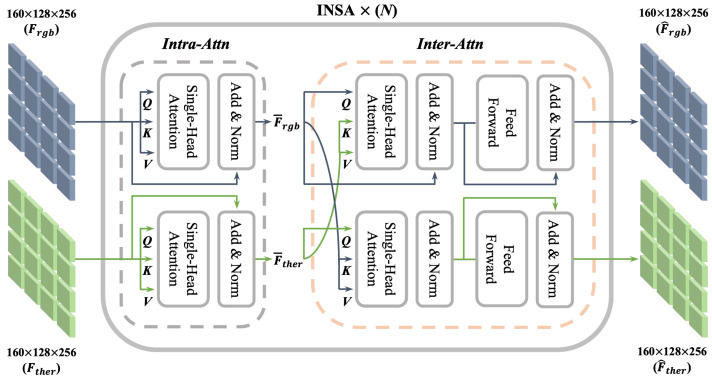
Proposed INtra-INter Spectral Attention (INSA) module. *Intra-Attn* and *Inter-Attn* indicate intra- and inter-spectral attention blocks, respectively. $F_{rgb}$ and $F_{ther}$ are the inputs of the INSA module. They are initially passed through *Intra-Attn* to enhance their representation, as indicated by ${\bar{F}}_{rgb}$ and ${\bar{F}}_{ther}$. Then, they are passed through *Inter-Attn* to capture the cross-modality interaction, resulting in the final outputs ${\hat{F}}_{rgb}$ and ${\hat{F}}_{ther}$, while also maintaining the feature map size of the input.

As illustrated in Figure 3, our INSA module comprises three major parts: an intra-spectral attention block, an inter-spectral attention block, and a feed-forward network. The intra-spectral attention and inter-spectral attention blocks are implemented in a standard self-attention and cross-attention manner. This is expressed here as Equation (3). Specifically, when two spectral feature maps, $F_{rgb}$ and $F_{ther}$, are input into the INSA module, the module initially applies intra-spectral attention blocks to the RGB and thermal feature maps independently. The goal of this is to enhance the modality-specific information within each set of spectral information by focusing on salient features and suppressing irrelevant features using an attention mechanism.

(7) $$\begin{array}{c} {\bar{F}}_{rgb}=Self-Attn\left(F_{rgb}\right),\;{\bar{F}}_{ther}=Self-Attn\left(F_{ther}\right) \end{array}$$

Next, inter-spectral attention blocks are employed to capture the cross-spectral interactions between ${\bar{F}}_{rgb}$ and ${\bar{F}}_{ther}$. This stage allows the model to understand the mutual relationship by aggregating complementary information across modalities. Finally, the processed feature maps are passed through a feed-forward network to refine the extracted information further.

(8) $$\begin{array}{cc} {\hat{F}}_{rgb},\;{\hat{F}}_{ther} & =INSA\left(F_{rgb},F_{ther}\right)=FFN\left(Cross-Attn\left({\bar{F}}_{rgb},{\bar{F}}_{ther}\right)\right) \end{array}$$

In Equation (8), *FFN* means the feed-forward network. The output feature maps of the INSA module contain both modality-specific and cross-modality complementary information, improving the feature fusion and detection performance.

To boost the efficiency, the INSA module also employs a shifted local window attention strategy for both intra- and inter-spectral attention. Specifically, we divide the entire input feature into K × K windows. The intra- and inter-attention processes are then applied independently within each window. To capture a broader context and avoid local optima, we also shift the window partition after processing one intra- and inter-spectral attention cycle. Consequently, this strategy reduces the computational complexity by focusing attention on shifted windows, thereby achieving significant efficiency gains compared to global attention mechanisms.

### 3.2. Analysis of Geometric Data Augmentation

The distribution of objects within an image plays a crucial role in the performance of anchor-based detectors, in which densely tiled anchor boxes are leveraged to localize the objects. Most methods resort to a general strategy that uniformly distributes anchors across the entire image under the assumption of equal importance for all image regions. However, most object detection datasets, such as Pascal-VOC [41] and MS-COCO [42], violate this assumption. In particular, multispectral pedestrian datasets such as KAIST [5] often suffer from an imbalance in pedestrian locations. This occurs because these datasets frequently include images taken in situations such as arterial roads, where the sidewalk and the road are clearly separated. These imbalances may cause the model to focus on regions where pedestrians frequently appear, thereby leading to the prediction of trivial solutions that only detect them around over-appearance areas such as these.

**Figure 4:**
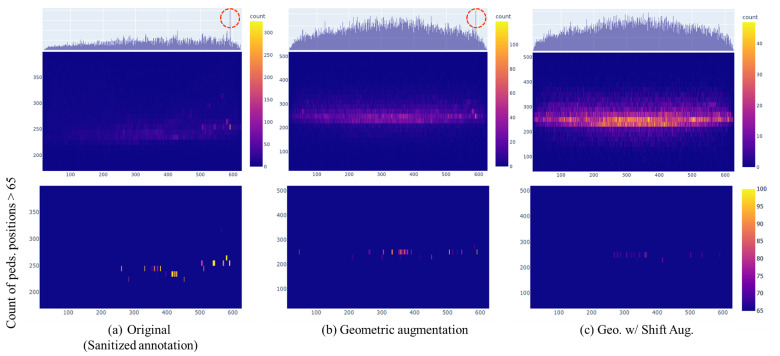
Histograms of pedestrian positions in the KAIST multispectral dataset, indicating the effects of different augmentations on pedestrian distribution. Note that we utilize the sanitized annotations of the training set to draw histograms. (**a**) Original: pedestrians heavily clustered in specific areas. (**b**) Geometric augmentation: distribution becomes more uniform, but some over-appearance persists. (**c**) Geo. w/Shift Aug: Combining geometric and shift augmentation significantly mitigates over-appearance, leading to a more uniform distribution. To visualize the phenomenon clearly, we highlight the over-appearance areas in row 1 with a red circle.

To mitigate this issue, numerous studies on pedestrian detection [11,14,15,43] employ common geometric augmentation techniques, such as cropping and flipping. As depicted in Figure 4, applying geometric data augmentation to a histogram of pedestrian locations in the KAIST dataset (shown in rows 1 and 2 of Figure 4a) results in a distribution that is relatively uniform, contrasting with the right-skewed distribution (shown in rows 1 and 2 of Figure 4b). However, as illustrated in the third row of Figure 4b, where we adjusted the pedestrian location count threshold in the histogram over 65 to highlight the phenomenon of concentrations in specific areas, only applying geometric augmentation continues to lead to pedestrian concentrations in certain regions (also shown in red circle of row 1).

To address the aforementioned problem, we design a shift augmentation, which performs translation transformation alongside geometric data augmentation. This method involves randomly shifting the image within a certain range in a direction opposite to the over-appearance area in the dataset. This serves to disperse the locations in over-appearance areas, thereby mitigating the concentration phenomenon.

As can be seen in Figure 4c, our shift augmentation strategy disperses the locations in over-appearance areas, alleviating the concentration phenomenon. This result demonstrates that our method can effectively alleviate the imbalance problem of pedestrian locations as opposed to applying only common geometric augmentation methods.

## 4. Experiments

### 4.1. Experimental Setup

#### 4.1.1. KAIST Dataset

The KAIST multispectral pedestrian dataset [5] consists of 95,328 fully overlapped RGB–thermal pairs in an urban environment. The provided ground truth consists of 103,128 pedestrian bounding boxes with pedestrians. In the experiment, we follow the standard criterion as *train02*, which samples frames such that a total of 25,076 images are used for training. For an evaluation, we also follow the standard evaluation criterion as *test20*, sampling 1 out of every 20 frames, such that all results are evaluated on 2252 frames consisting of 1455 day images and 797 night images. Additionally, we conducted experiments with different driving scenes that were divided into three subsets: *Campus* (set06, set09) with 823 frames, *Road* (set07, set10) with 850 frames, and *Downtown* (set 08, set11) with 579 frames. Note that we use paired annotations for training [11] and sanitized annotations for the evaluation [16]. This is the standard criterion for a fair comparison with recent works.

#### 4.1.2. LLVIP Dataset

The LLVIP dataset [10] is a recently released multispectral pedestrian dataset in low-light vision environments. This dataset is composed of RGB-IR pairs consisting of 30,976 images, or 15,488 pairs, collected under challenging environments such as insufficient illumination conditions or heavily obscured. Contrasted with the KAIST dataset, which relies on systematic configuration for alignment, the LLVIP dataset was captured using a stereo configuration binocular camera. However, strict spatial and temporal alignment was achieved through post-processing image registration. In the experiment, we adhere to the established protocol of prior studies [10,19] utilizing 12,025 and 3463 image pairs for training and testing, respectively.

### 4.2. Implementation Details

We conducted experiments using NVIDIA A100 GPUs with PyTorch. Our baseline network was modified from a fusion architecture [23] using SSD [43]. We employed batch normalized VGG-16 as a backbone, which was initialized with ImageNet pre-trained weights up to *conv3* before the fusion stage. We also reduced the model complexity by modifying the auxiliary network of SSD [43] by removing the *conv11* layer. For training, we utilized *Momentum Stochastic Gradient Descent (Momentum SGD)* with an initial learning rate, momentum, and weight decay of $10^{-4}$, 0.9, and $5\times 10^{-4}$, respectively. The batch size was set to 8, and both training and evaluation input images were resized to 640 (W) × 512 (H). We utilize data augmentation in the following order: the proposed shift augmentation, a spectral-independent horizontal flip, and a random resized crop, with the probability of applying each transformation set to 0.3, 0.5, and 0.5, respectively. Specifically, shift augmentation randomly shifts the multispectral images along the x-axis by an integer value in the range of 0 to 20. While shift augmentation and random resized crop are applied to both RGB and thermal images, a spectral-independent horizontal flip is processed separately on each modality image. We utilized a general detection loss that consists of the classification and localization loss and employed the multi-label classification loss [15] to enable the model to learn the modality-inherent features. Finally, the network was trained for 40 epochs with a batch size of 8.

### 4.3. Evaluation Metric

We use the standard log-average miss rate (LAMR), the most popular metric for pedestrian detection tasks, as a representative metric, sampled for a false positive rate per image (FPPI) in the range [$10^{-2},10^{0}$], as proposed by Dollar et al. [44]. This metric is more appropriate for commercial solutions because it focuses on areas of high accuracy rather than areas of low accuracy.

### 4.4. Comparison with State-of-the-Art Multispectral Pedestrian Detection Methods

#### 4.4.1. KAIST Dataset

To validate the effectiveness of the proposed method, we compare it with state-of-the-art multispectral pedestrian detectors, in this case ACF [45], Halfway Fusion [23], MSDS-RCNN [16], AR-CNN [11], MBNet [18], MLPD [15], CFT [19], GAFF [13], and CFR [17]. Table 1 shows the detection results of our method and of the state-of-the-art detectors on the KAIST dataset. In ALL, which included both day and night, we achieved a miss rate of 5.50%, which is 0.46% higher than the previous best method, CFR [17]. From these results, our method demonstrates efficiency as a fusion method that performs complementary information exchanges while preserving the unique characteristics of the two modalities depending on day and night scenes. Furthermore, despite the different pedestrian distributions on *Campus*, *Road*, and *Downtown*, the proposed method shows the best performance on *Road* and *Downtown* while maintaining competitive accuracy on *Campus* (7.45 from CFR vs. 7.64 from ours).

**Figure 5:**
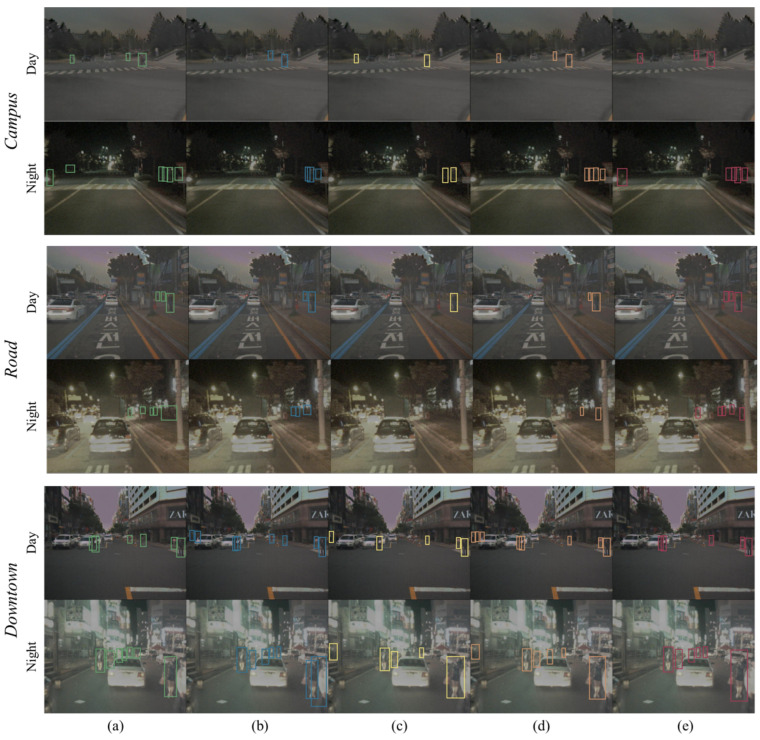
Qualitative results of the proposed method on the KAIST dataset. The comparison results demonstrate that our method (**e**) effectively alleviates the concentrated distribution and efficiently learns the mutual spectral relationships. We compared the ground truth-(**a**) with three state-of-the-art multispectral pedestrian detectors: (**b**) MLPD [15], (**c**) GAFF [13], and (**d**) CFR [17]. For comparison according to the driving environment, the KAIST dataset is composed of *Campus*, *Road*, and *Downtown* from the top in units of two rows. The first row shows Day, the second row shows Night, and this is repeated for the remaining row groups. Following the standard evaluation protocol [5], we exclude predict boxes with a height is 55 or less.

Figure 5 illustrates the qualitative results of our method in comparison with MLPD [15], GAFF [13], and CFR [17] on the KAIST dataset. In comparison with these other methods, our method shows better detection results by explicitly detecting ambiguous targets during both the day and night. In addition, while other methods tend to produce more false positive results on the left side due to the right-skewed pedestrian distributions, our method shows reliable detection results by alleviating the over-appearance of pedestrians using the shift augmentation strategy.

#### 4.4.2. LLVIP Dataset

To further demonstrate the generality of the proposed method, we conducted experiments on the LLVIP dataset. Note that as mentioned in Section 1, LLVIP has a more uniform distribution compared to KAIST, but it still exhibits an over-appearance region on the right side of the images. Therefore, we conducted experiments with an identical setup as in the KAIST and compared it with state-of-the-art detectors, in this case, Yolo [46], FBCNet [47], and CFT [19]. Table 2 shows the detection results of the proposed method and of the state-of-the-art detectors on the LLVIP dataset. In the experimental results, we achieved a miss rate of 4.43%, which is 0.97% higher than the previous best method, CFT [19]. It is interesting to note that when comparing with and without shift augmentation, the miss rate is 5.64 without shift augmentation, which is 0.24% higher than CFT. However, the miss rate decreases by 1.21% after applying shift augmentation, which is 0.97% lower than CFT. From these results, our method also demonstrates its effectiveness on other benchmarks with state-of-the-art performance.

### 4.5. Ablation Study

#### 4.5.1. Effects of INtra-INter Spectral Attention

We ablate the components of the proposed INtra-INter Spectral Attention (INSA) module in Table 3. First, we chose the halfway SSD architecture with a halfway fusion mechanism that performs multispectral feature fusion by directly applying intermediate feature maps to a weighted sum as the baseline model. Note that we evaluate the model that was trained with and without our shift augmentation strategy to focus on the effectiveness of the INSA components.

As shown in Table 3, inter-spectral attention, which allows the model to focus on relevant regions between multispectral images, achieves satisfactory results compared to the baseline (7.50 → 6.66 for the miss rate). This suggests that inter-spectral attention effectively captures complementary information across different spectral images. Moreover, intra-spectral attention can improve the detection performance (7.50 → 6.81 for the miss rate) by enhancing the modality-inherent information within each spectral image. This result indicates that enhancing the individual spectral features is equally important considering the mutual relationships across modalities in multispectral pedestrian detection. Lastly, as we carefully designed the INSA module that initially enhances the individual spectral features and then captures the mutual relationship among them, our model results in the best performance, as shown in the last row of Table 3.

#### 4.5.2. Hyperparameters in INSA

We ablate the hyperparameters of the proposed INSA module in Table 4. As shown in Table 4, we found that the INSA module with two iterations, where the output of the first INSA module is fed back to the intra-attention block of the second INSA module, achieves the best detection performance (6.12%). Also, we ablate to find the optimal number of input tokens for the INSA module in Table 4. Performance results show that using 16 tokens, i.e., dividing the input feature map into 4 × 4 patches, achieves the best detection performance (6.12%). In accordance with the optimal hyperparameters identified in the ablation studies, we utilized 16 patches and two iterations of INSA modules for all experiments.

#### 4.5.3. The Impact of Performance from Geo and Shift Augmentation

As discussed in Section 3.2, the pedestrian distribution within the images significantly affects the model training and accuracy. To investigate how the impact of the pedestrian distribution generalizes across models, we assess the detection performance capabilities of SSD with halfway fusion as well as the proposed framework with the INSA module in Table 5.

When the geo is marked, the results with both Baseline and INSA show a superior performance gain compared to models without geo. These results demonstrate that the imbalanced distribution of pedestrians significantly reduces the detection performance, whereas geometric transformation mitigates this impact effectively by augmenting the training data. It is interesting to note that Baseline with geo shows impressive results on Road, as shown in the third row of Table 5 (1.93 miss rate). However, the performance on the test datasets overall (ALL) fell short of the baseline when using both geo and shift (7.50 miss rate with geo only vs. 7.03 miss rate with geo and shift). This performance gap is attributed to Baseline with geo and shift achieving better performance on Campus and Downtown, where the pedestrian distributions are more diverse compared to that of Road.

We find that in the third row of each method in Table 5, applied shift augmentation alone outperforms without augmentation but underperforms when applied to geo alone. This is because geo utilizes diverse transformations, such as cropping and flipping, which have a more uniform distribution than shift augmentation alone. However, we focus on the significant performance improvement when applying shift augmentation alone in cases of road scenes where the pedestrians are extremely skewed to the right of the image. This is because even when using shift augmentation alone, it mitigates over-appearance regions.

These experimental results, along with the observation that the overall miss rate is lowest when both geo and shift augmentation are applied, we confirm that shift augmentation plays a complementary role to geo. In other words, shift augmentation helps address the persistent issue of pedestrian over-appearance in certain areas even after applying geo. As a result, shift augmentation encourages the model to learn broader pedestrian features and ultimately achieve better generalization detection performance.

#### 4.5.4. Hyperparameters in Shift Augmentation

In Table 6, we compare the performance relative to the random movement range and probability of shift augmentation. We note that positive and negative values represent shifting an image to the right and left side, respectively. The proposed model exhibits an optimal performance miss rate of 5.50% at Δ−20 with an application probability rate of *p* = 0.3. When the image is shifted in the same rightward direction as in the KAIST dataset, which has a distribution of pedestrians concentrated on the right, the performance decreases compared to the performance without shift augmentation. This demonstrates that shift augmentation can alter the distribution of pedestrians within the dataset by moving the image. Moreover, this implies that shifting the image away from the concentrated distribution can improve detection performance by mitigating the over-appearance issue in regions.

## 5. Conclusions

In this paper, we design INSANet to address the limitations of CNN-based multispectral fusion strategies, which mainly focus on local feature interactions due to their limited receptive field. More specifically, our attention-based fusion module effectively integrates intra- and inter-modality information, overcoming the limitations of existing strategies that prevent the corresponding models from interpreting relationships across modalities. Furthermore, we investigate the effect of data augmentation to address the imbalanced over-appearance pedestrian location distributions in training data. With our contributions, including the INSA module and shift augmentation, our model could learn the representation of pedestrians at various locations and the complementary information between multispectral images. In the experimental section, we demonstrate that the proposed method outperforms the recent state-of-the-art methods in terms of detection accuracy on the KAIST multispectral dataset [5]. Although our shift augmentation method can effectively improve the performance of pedestrian detection on the dataset tested here, the optimal shift range may vary depending on the dataset used. To address this issue, our future work will focus on designing a generalizable augmentation framework that automatically selects the optimal hyperparameters. We believe that this will lead to the development of an effective multispectral pedestrian detection framework applicable to a wider range of real-world scenarios.

## Figures and Tables

**Table 1:** Benchmark of the pedestrian detection task on the KAIST dataset. † is the re-implemented performance with the proposed fusion method. The highest performance is highlighted in bold, while the second-highest performance is underlined.

Method	Miss Rate (%)					
	ALL	DAY	NIGHT	*Campus*	*Road*	*Downtown*
ACF [45]	47.32	42.57	56.17	16.50	6.68	18.45
Halfway Fusion [23]	25.75	24.88	26.59	-	-	-
MSDS-RCNN [16]	11.34	10.53	12.94	11.26	3.60	14.80
AR-CNN [11]	9.34	9.94	8.38	11.73	3.38	11.73
$Halfway\;Fusion{\;}^{†}$	8.31	8.36	8.27	10.80	3.74	11.00
MBNet [18]	8.13	8.28	7.86	10.65	4.25	9.18
MLPD [15]	7.58	7.95	6.95	9.21	5.04	9.32
ICAFusion [20]	7.17	6.82	7.85	-	-	-
$CFT{\;}^{†}$ [19]	6.75	7.76	4.59	9.45	3.47	8.72
GAFF [13]	6.48	8.35	**3.46**	7.95	3.70	8.35
CFR [17]	5.96	8.35	**3.46**	**7.45**	4.10	7.25
${\mathrm{Ours}}_{\left(w/o\;shift\right)}$	6.12	7.19	4.37	9.05	3.24	7.25
${\mathrm{Ours}}_{\left(w/shift\right)}$	**5.50**	**6.29**	4.20	7.64	**3.06**	**6.72**

**Table 2:** Benchmark of the pedestrian detection task on the LLVIP dataset. The highest performance is highlighted in bold, while the second-highest performance is underlined.

Method	Spectral	Miss Rate (%)
Yolov3 [46]	visible	37.70
	infrared	19.73
Yolov5 [10]	visible	22.59
	infrared	10.66
FBCNet [47]	visible	19.78
	infrared	7.98
MLPD [15]	multi	6.01
CFT [19]		5.40
${\mathrm{Ours}}_{\left(w/o\;shift\right)}$	multi	5.64
${\mathrm{Ours}}_{\left(w/shift\right)}$		**4.43**

**Table 3:** Attention-wise ablation for the proposed INtra-INter Spectral Attention module. Self refers to models using only self-attention, and Cross refers to models using only cross-attention. The highest performance is highlighted in bold.

Attention		Miss Rate (%)
Intra (Self)	Inter (Cross)	ALL
-	-	7.50
√	-	6.81
-	√	6.66
√	√	**6.12**

**Table 4:** Comparisons of performance results on the proposed INtra-INter Spectral Attention module hyperparameters. The highest performance is highlighted in bold.

**Iterations of Modules *N***	
N	**MR** (%)
1	6.16
2	**6.12**
4	6.20
**Number of Patches**	
**Patch**	**MR** (%)
8	6.61
16	**6.12**
32	6.88

**Table 5:** Ablation study of shift augmentation. geo refers to geometric transformations that use random flips and random crops with a given probability. Baseline is a detector that applies a weighted sum for both modalities without using the INSA module. When both geo and shift are not marked, the model is only trained using color jitter augmentation. The highest performance is highlighted in bold.

Method	Aug.		Miss Rate (%)					
	Geo	Shift	ALL	DAY	NIGHT	*Campus*	*Road*	*Downtown*
Baseline	-	-	11.50	13.98	6.83	14.82	8.22	13.73
	-	√	10.58	12.29	7.11	13.67	3.17	13.59
	√	-	7.50	8.84	**4.70**	11.06	**1.93**	9.18
	√	√	**7.03**	**7.85**	5.38	**9.40**	3.29	**8.81**
INSA_(Ours)_	-	-	10.11	11.18	7.86	12.74	6.04	12.36
	-	√	9.30	10.42	7.35	11.89	**2.92**	12.24
	√	-	6.12	7.19	4.37	9.05	3.24	7.25
	√	√	**5.50**	**6.29**	**4.20**	**7.64**	3.06	**6.72**

**Table 6:** Performance comparison according to the hyperparameters of shift augmentation. In the random movement range Δ, negative values indicate a move to the left and positive values indicate a move to the right. The probability *p* represents the probability that augmentation will be applied. The bold performance is highest performance and underlined performance is the result without applying shift, and if the performance improves compared to this, it is indicated in italics.

		Miss Rate (%)						
		Δ−30	Δ−20	Δ−10	0	Δ10	Δ20	Δ30
*p*	1.0	7.26	6.96	6.87	6.12 (*p* = 0)	7.18	7.49	7.57
	0.7	6.71	*6.07*	6.63		6.80	6.70	7.61
	0.5	6.76	*5.85*	*5.93*		7.00	7.21	7.16
	0.3	6.33	***5.50***	*5.95*		6.27	6.22	6.93

## Data Availability

The data presented in this study are available on request from the corresponding author. The provided data can be only used for nonprofit purposes.
